# Supplementary material for: Complexome profiling of the Chlamydomonas psb28 mutant reveals TEF5 as an early PSII assembly factor
Source: Plant Cell. 2025 Mar 17;37(6):koaf055. doi: 10.1093/plcell/koaf055 (PMC12164590; doi:10.1093/plcell/koaf055)
Supplement: koaf055_Supplementary_Data [file koaf055_supplementary_data.zip › psb28_interactive.zip!/plots/intensity/qualityPlots/peptides/_Cre16.g688800.t1.2.html]

### Cre16.g688800.t1.2

Trivial name:   
SeqCoverage [median]: 21.20  
Mass: 20.64 kDa  
  
 

| Sequence | MissedCleavage | Mass [Da] | wtIntensity | muIntensity | Score |
| --- | --- | --- | --- | --- | --- |
| AEQADADKAGAER | 1 | 1330.61 | 13633.22 | 12944.58 | 150.78 |
| APDAAGAAK | 0 | 770.39 | 6788.41 | 11277.14 | 157.58 |
| MLQQMGYR | 0 | 1025.48 | 4049.32 | 4166.27 | 99.92 |
| STYAGSSLSAK | 0 | 1070.52 | 3509.21 | 9801.16 | 119.22 |
|  |  |  | 27980.2 | 38189.1 |  |
